# Spatial and temporal transcriptome changes occurring during flower opening and senescence of the ephemeral hibiscus flower, *Hibiscus rosa-sinensis*

**DOI:** 10.1093/jxb/erw295

**Published:** 2016-09-03

**Authors:** Alice Trivellini, Giacomo Cocetta, Donald A. Hunter, Paolo Vernieri, Antonio Ferrante

**Affiliations:** ^1^Institute of Life Science, Scuola Superiore Sant’Anna, Pisa, Italy; ^2^Department of Agricultural and Environmental Sciences, Università degli Studi Milano, Milan, Italy; ^3^The New Zealand Institute for Plant & Food Research Limited, Palmerston North, New Zealand; ^4^Department of Agriculture, Food and Environment, Università degli Studi di Pisa, Pisa, Italy

**Keywords:** 454 sequencing, aquaporins, cell wall, light and circadian clock signalling, microarray, ovary, PCD, petal, style-stigma plus stamens.

## Abstract

Flowers are complex systems whose vegetative and sexual structures initiate and die in a synchronous manner. The rapidity of this process varies widely in flowers, with some lasting for months while others such as *Hibiscus rosa-sinensis* survive for only a day. The genetic regulation underlying these differences is unclear. To identify key genes and pathways that coordinate floral organ senescence of ephemeral flowers, we identified transcripts in *H. rosa-sinensis* floral organs by 454 sequencing. During development, 2053 transcripts increased and 2135 decreased significantly in abundance. The senescence of the flower was associated with increased abundance of many hydrolytic genes, including aspartic and cysteine proteases, vacuolar processing enzymes, and nucleases. Pathway analysis suggested that transcripts altering significantly in abundance were enriched in functions related to cell wall-, aquaporin-, light/circadian clock-, autophagy-, and calcium-related genes. Finding enrichment in light/circadian clock-related genes fits well with the observation that hibiscus floral development is highly synchronized with light and the hypothesis that ageing/senescence of the flower is orchestrated by a molecular clock. Further study of these genes will provide novel insight into how the molecular clock is able to regulate the timing of programmed cell death in tissues.

## Highlight

Pathway analysis suggests that identified temporal and spatial transcriptomic changes associated with senescence of the short-lived hibiscus flower are regulated by light/circadian clock-, aquaporin-, cell wall-, and calcium-related gene families.

## Introduction

Flowers in the Angiospermae have been shaped over time by natural selection into a diversity of sizes, shapes, and colours. Just as these visual attributes have been continually influenced by environmental and other selection pressures, so too has the timing and duration of their flowering and the rapidity at which each individual flower initiates and senesces.

Senescence of flowers involves sequential degeneration of all flower parts except (if pollination is successful) the ovary of the carpel, which becomes a nutrient sink that develops to maximize seed viability and dispersal. Among the flower organs, petals are the first to senesce. This can be rapid, particularly following pollination, and is thought to increase the efficacy of the pollinators by signalling which flowers have previously been visited (Stead, 1992; O’Neill, 1993, 1997; van Doorn, 1997). Various studies have revealed that flower senescence can visually manifest in different ways (Woltering and van Doorn, 1988; van Doorn and Woltering 2008). Some flowers wilt and abscise, others abscise before they wilt (Woltering and van Doorn, 1988; Rogers, 2012). There are many biochemical and molecular changes that underlie these different senescence responses.

Over the years, all the major hormone groups have been explored for their effect on senescence. Of these, ethylene arguably is the most potent senescence initiator for many flowers (Woltering and van Doorn, 1988). In ethylene-sensitive flowers, the hormone can cause wilting and/or abscission. Many, but not all, ethylene-sensitive flowers show an age-related increase in the production of the hormone. Some exceptions are daffodil (*Narcissus* spp.) and *Campanula* flowers, which, although very sensitive to ethylene, produce negligible amounts of the hormone unless pollinated (Hunter *et al.*, 2004). Consequently, inhibitors of ethylene action minimally affect the timing of their age-related senescence, but do delay their pollination-induced senescence. The two key enzymes in ethylene biosynthesis are 1-aminocyclopropane-1-carboxylic acid (ACC) synthase and oxidase and their suppression by antisense technology has been successful in prolonging floral display life (Savin *et al.*, 1995). Ethylene is produced by all plant organs but whether it initiates senescence depends on the competency of the tissues to respond to the hormone. This senescence competency involves age-related changes, as can be seen by the inability of the hormone to initiate senescence in young floral buds (Olsen *et al.*, 2015). Transcription factors that potentially drive ethylene-induced flower senescence responses have been identified. These include ETHYLENE INSENSITIVE3 (EIN3)-like genes in carnation (*Dianthus caryophyllus*; Iordachescu and Verlinden, 2005) and ETHYLENE-RESPONSIVE ELEMENT BINDING FACTORS (ERFs) in daffodils (Hunter *et al.*, 2002) and petunia (*Petunia hybrida*; Liu *et al.*, 2010). More recently, virus-induced gene silencing and overexpression studies have revealed a basic helix-loop-helix transcription factor, PhFBH4, that positively controls the timing of petunia flower senescence, likely by regulating induction of the ethylene biosynthetic pathway (Yin *et al.*, 2015).

Abscisic acid (ABA) has also been reported to accelerate senescence in flowers and, like ethylene, the effect of ABA varies from species to species and from cultivar to cultivar; it may also be governed by developmental stage (Rogers, 2013). The importance of ABA can be confounded by its interaction with ethylene. ABA induces flower senescence in miniature roses (*Rosa hybrida*), in part through the induction of ethylene production, an effect that is delayed by the ethylene action inhibitor 1-methycyclopropane (1-MCP; Müller *et al.*, 1999). In daffodils ABA accelerates senescence independently of ethylene, as demonstrated by the early accumulation of senescence-associated transcripts that was not prevented by pre-treatment of the flowers with 1-MCP (Hunter *et al.*, 2004). In *Hibiscus* spp., ABA and ethylene both accelerate floral senescence, with ABA doing so despite lowering the concentrations of ethylene in the petal tissue (Trivellini *et al.*, 2011*a*, 2011*b*). Endogenously produced ABA is thought to be the key hormone regulating flower senescence in ethylene-independent flowers such as daylily (*Hemerocallis* spp.), with exogenously supplied ABA accelerating senescence-associated events such as a loss of membrane permeability and lipid peroxidation (Panavas *et al.*, 1998).

Cytokinins and auxins can also control floral longevity. Chang *et al.* (2003) found that transgenic petunia which overproduce cytokinins had longer flower life compared with the wild-type controls, and a similar effect was found when the hormone was applied exogenously (Trivellini *et al.*, 2015*a*). The senescence of wallflowers is delayed by 2 days when they are treated with the cytokinin oxidase inhibitor 6-methyl purine (Price *et al.*, 2008). In Arabidopsis, auxin response factors (ARFs) control flower development and senescence. For example, ARF2 positively regulates stamen length, floral organ abscission, flowering time, and silique dehiscence (Ellis *et al.*, 2005).

Reactive oxygen species (ROS) are produced in increasing amounts during senescence and their action is counteracted by endogenous defence mechanisms (Rogers, 2012). Among these, plant pigments can limit ROS formation, delay senescence symptoms, and prolong flower longevity. This is possible because many of these pigments, such as anthocyanins and carotenoids, are potent antioxidants and protect the cells from the damage caused by senescence. Higher anthocyanin content is associated with longer flower life in petunia (Ferrante *et al.*, 2006).

The senescence of the individual whorls of flowers is coordinated, with some flower organs appearing to determine the longevity of other organs. For example, in carnations the ovary has a major role in determining the timing of petal senescence, as shown by the fact that removing the ovaries lengthens the display life (ten Have and Woltering, 1997; Shibuya *et al.*, 2000; Jones, 2013). These results are connected with the reproduction cycle of plants and in particular with pollination (O’Neill *et al.*, 1993). When flowers senesce early in response to pollination it is invariably due to the increased production of ethylene by the flower, often by the gynoecium. By contrast, in flowers that are ephemeral (lasting a day or less), pollination typically does not affect floral longevity (van Doorn, 1997).

Although progress has been made in understanding some of the regulation of flower petal senescence, detailed knowledge of the molecular control of all floral organs during flower senescence, particularly that of ephemeral flowers, is still lacking. To increase our understanding of this phenomena, we performed transcriptional profiling on different plant organs of *Hibiscus rosa-sinensis* flowers at different stages of their development. Hibiscus flowers are particularly suited for this because of their short-lived nature (lasting only 24h) and because their different developmental stages can be accurately and precisely identified based on key changes associated with maturation of their sexual organs (i.e. bud growth, petal opening, anther maturation, pollination, petal wilting, ovary maturation after pollination) (Trivellini *et al.*, 2011*b*, 2015*b*).

Here we report how the significantly enriched biological themes identified by transcriptome profiling change in the different floral organ tissues of the hibiscus flower as it transitions from a bud through to a senescent flower. We also contextualize the gene changes by placing them into the functional categories ‘Trigger and signalling’, ‘Transcriptional regulation’, ‘Coordination’, and ‘Execution’, as have been defined for developmentally controlled programmed cell death by Van Hautegem *et al.* (2015). By identifying these transcriptome responses, we have obtained a detailed and clear insight into the dynamic spatial and temporal changes associated with the life of an ephemeral flower.

## Materials and methods

### Plant materials and growth conditions

Flowers of *H. rosa*-*sinensis* L. ‘La France’ plants were used for the transcriptome sequencing. The plants were grown in a greenhouse under natural environmental conditions and detached flowers were used as the experimental material. All experiments were performed between 15 May and 30 September. Flowers were harvested between 7:30 am and 8:00 am either on the morning of flower opening (open flowers, OF), the morning of the day before opening (bud flowers, B), or the morning of the day after opening (senescent flowers, SF)as described in Trivellini *et al.* (2011*b*).

### RNA isolation and double-strand cDNA preparation for 454 sequencing

Tissues were ground under liquid nitrogen before extraction of total RNA using the Spectrum Plant Total RNA Kit with on-column DNase-treatment (Sigma, Italy) according to the manufacturer’s instructions. RNA concentration and integrity were assessed with a NanoDrop N-1000 spectrophotometer (ThermoFisher Scientific) and standard formaldehyde agarose gel electrophoresis. First-strand cDNA synthesis was performed using the SMART cDNA Synthesis Kit (Clontech Laboratories) and SuperScript III Reverse Transcriptase (Life Technologies).

Double-stranded cDNA was amplified by long-distance PCR using the Advantage^®^ 2 PCR Enzyme System (Clontech Laboratories). Amplification was performed in a thermal cycler (Applied Biosystems) with the following PCR parameters: 95°C for 1min; 16 cycles of 95°C for 15s and 65°C for 30s; 68°C for 6min; and 4°C for 45min. The double-stranded cDNA was resolved in a 1.1% agarose gel containing ethidium bromide and the cDNA synthesis verified by visualization under UV light.

Samples were sequenced using the 454 GS-FLX instrument according to the manufacturer’s instructions (Roche). Transcriptome data have been published in the GenBank database [PRJNA325155].

### Microarrays analysis: amino allyl antisense RNA synthesis, labelling, and hybridization

Total RNA of complete flowers containing all parts (petals, style-stigma + stamen [S-S+S]) was amplified using the Amino Allyl MessageAmp II aRNA Amplification Kit (Ambion) to obtain amino allyl antisense RNA (aaRNA) following the method of Eberwine *et al.* (1992). Briefly: mRNA was reverse-transcribed in a single strand of cDNA; after second strand synthesis (in the second round of amplification), cDNA was *in vitro* transcribed in aaRNA including amino allyl modified nucleotides (aaUTP). Both double-stranded DNA and aaRNA underwent a purification step using columns provided with the kit.

Labelling was performed using NHS ester Cy3 or Cy5 dyes (Amersham Biosciences) able to react with the modified RNA (Yang *et al.*, 2002). mRNA quality was checked with RNA 6000 nano chip assays (Agilent Technologies). At least 5 μg of mRNA for each sample was labelled and purified using the columns. Equal amounts (0.825 μg) of labelled specimens from B vs OF, B vs SF, and OF vs SF were put together, fragmented, and hybridized to oligonucleotide 60bp glass arrays representing the hibiscus transcriptome. An Agilent 4x44K cDNA-chip (hib-chip) was commissioned to facilitate flower transcriptome analysis and senescence studies. Each slide contained four microarrays. The probes were 60bp long and five replicates of each probe were randomly located on the microarray slide. All steps were performed using the *In Situ* Hybridization Kit Plus (Agilent Technologies) following the 60-mer oligo microarray processing protocol (Agilent Technologies). Slides were washed using the Agilent wash procedure and scanned with the dual-laser microarray scanner Agilent G2505B. For each sample, a dye-swap replicate was performed. The gene expression of each sample was normalized and the background corrected by linear modelling using a moderated *t*-statistic as implemented in the limma package for the R statistical environment (v.2.9.2). The design of the chips used is shown in Supplementary Table S1. The microarray results represent an average of three independent biological replicates for each flower organ.

### Gene-annotation enrichment analysis

Biological interpretation of gene sets was accomplished using The Database for Annotation, Visualization and Integrated Discovery (DAVID 6.7; Huang *et al.*, 2009, http://david.abcc.ncifcrf.gov/home.jsp). DAVID identifies enriched biological themes within the gene sets using over 40 annotation categories, including gene ontology (GO) terms, protein–protein interactions, and protein functional domains. The cluster enrichment score was obtained using the Functional Annotation Clustering (FAC) and Gene Functional Classification tools, which cluster functionally related annotations into groups and ranks them in importance with an enrichment score. An enrichment score of 1.3 for a cluster is equivalent to non-log scale 0.05, and therefore scores ≥1.3 are considered enriched. The Functional Annotation Chart was used to give an overall idea of gene distributions among terms (Huang *et al.*, 2009).

### Validation of genes using quantitative PCR

The microarray profiling results were validated using quantitative reverse transcription (RT)-PCR measurements on chosen ethylene biosynthesis genes in different flowers organs (petals and S-S+S) at the different development stages B, OF, and SF. Primer sequences and quantitative PCR conditions are as reported previously (Trivellini *et al.*, 2011*a*). Gene transcript abundance changes showed the same trends as observed in the microarray described above.

## Results and discussion

### Spatial and temporal transcriptome profiling of the hibiscus flower during development and senescence

**Fig. 1.**
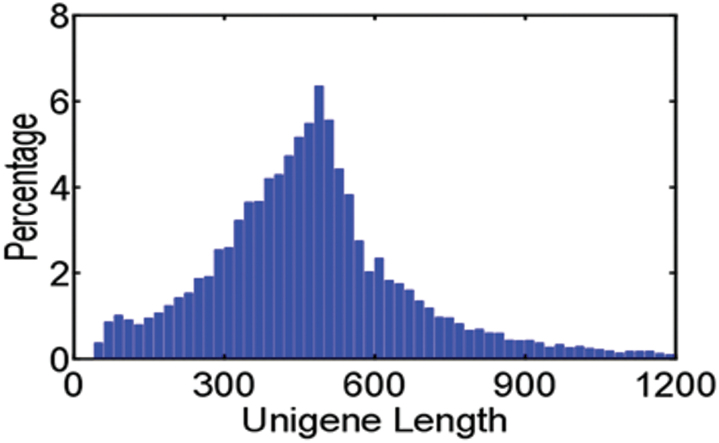
Sequence length distribution of unigenes in *H. rosa-sinensis* flowers.

To unravel the transcription-based regulatory network occurring in *Hibiscus rosa-sinensis* L. flowers as they develop and senesce, we performed next-generation 454 pyrosequencing in combination with microarray profiling. The 454 GS-FLX sequencing was performed using double-stranded cDNA derived from two tissue samples (the flower bud, and a combination of fully opened and incipiently senescent flowers). After removing low-quality regions and adaptors, we obtained 257,598 high-quality ESTs with an average length of 319bp and a total length of 82.2Mb. Of these ESTs, 137,409 were from the bud stage and 120,189 from fully opened/senescent flowers (Table 1). After clustering and assembly using Phrap (http://www.phrap.org/phredphrap/phrap.html), 257,598 sequences were incorporated into 23,622 contigs, leaving 7326 singlets, for a total of 30,988 unigenes. Of these unigenes, 23,058 were from bud and 18,102 from opened/senescent flowers (Table 1). The unigenes had an average length of 487.3bp (Fig. 1).

**Table 1.** Statistics of *H. rosa-sinensis* flower ESTs generated by the 454 GS-FLX platform

	No. of reads	Average read length (bp)	Median read length (bp)	Total bases (bp)	No. of unigenes
**Bud**	137409	339.11	381	46596840	23058
**Opened/senescent flower**	120189	296.38	318	35621337	18102
**Total**	257598	319.2	351	82218177	30988

For annotation, the 30,988 unigenes were searched against the NCBI non-redundant (Nr) database using BlastX; the NCBI nucleotide (Nt) database using BlastN, setting a cut-off E-value of 10^–5^; UniProtKB/Swiss-Prot (SwissProt), Kyoto Encyclopedia of Genes and Genomes (KEGG), and Clusters of Orthologous Groups (COG). The annotated sequences are reported in Supplementary Table S1. Based on sequence homology, we successfully assigned annotations to 20,547 unigenes from the NCBI Nr protein database, 16,071, from SwissProt, and 20,278 from KEGG (Table 2).

**Table 2.** Statistics of annotation results for *H. rosa-sinensis* unigenes

	Total unigenes	Nt	Nr	SwissProt	KEGG	COG
**Bud**	23058	20575	16302	13184	16233	5697
**Opened/senescent flower**	18102	15792	11151	8061	10744	4033
**Total**	30988	27195	20547	16071	20278	6977
**E cut-off**		1.00E^−05^	1.00E^−05^	1.00E^−10^	1.00E^−10^	1.00E^−10^

**Fig. 2.**
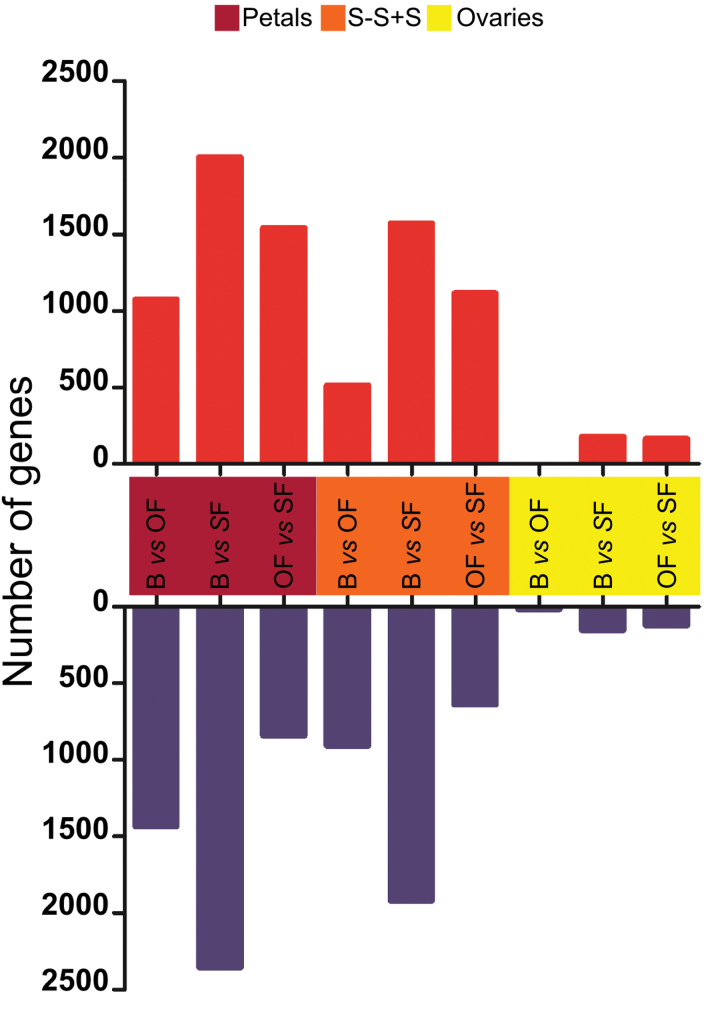
Number of genes differentially expressed in different floral tissues (petals, style-stima+stamen [S-S+S] and ovaries) during flower development. The comparisons were bud versus open flower (B vs OF), bud versus senescent flower (B vs SF), and opened flower versus senescent flower (OF vs SF). Red and blue indicate upregulation and downregulation, respectively.

Transcriptome analysis was performed on isolated flower tissues (petals, ovary, S-S+S) harvested at different developmental stages (B, OF, and SF) as described previously in Trivellini *et al.* (2011*a*, *b*). The following comparisons were performed: B vs OF, B vs SF, and OF vs SF. Profiling indicated that a large number of transcripts (7110) were differentially expressed (Fig. 2). Those showing a change of 2-fold or more with a significant *P* value (*P* ≤ 0.05) are indicated in Supplementary Table S2.

**Fig. 3.**
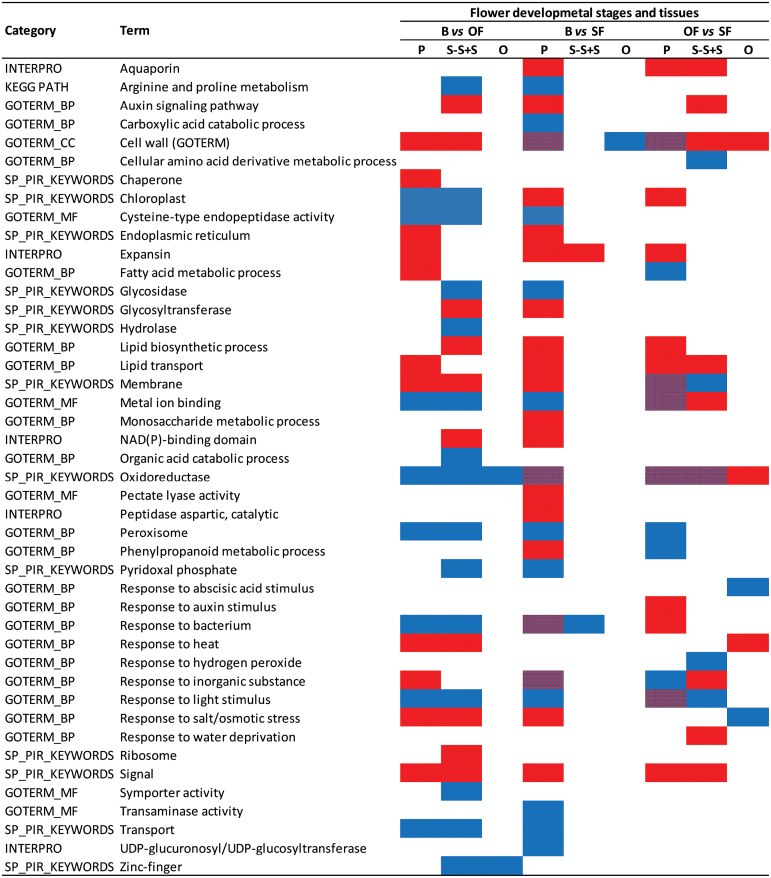
Over-representation analysis of gene terms upregulated and/or downregulated by more than 2-fold between flower developmental stages (bud versus opened flowers, B vs OF; buds versus senescent flowers, B vs SF; opened flowers versus senescent flowers, OF vs SF) and tissues (petal, P; style-stigma+stamens, S-S+S; ovary, O) according to functional categories (DAVID). Selected significantly enriched functional annotation clusters are shown together with the term(s) most representative of each cluster. Only those term(s) showing a cluster enrichment scores >1.3, a significant *P* ≤ 0.05 after correction for multiple testing by the Benjamini-Hochberg method, and a count ≥10 are shown. Red, blue, and overlap red/blue, represent enriched terms in the upregulated, downregulated, and both up- and downregulated gene sets, respectively.

DAVID (http://david.abcc.ncifcrf.gov/, Huang *et al.*, 2009) provided a complementary statistical overview into the enrichment of pathways and gene function based on GO and other functional annotation data (Fig. 3). Of the annotation clusters found by DAVID to change significantly, the category ‘cell wall’ was the most enriched and was found in almost all up- and downregulated gene sets (Fig. 3; Supplementary Table S3). This fits well with the documented profound structural and morphological changes that occur in petal cell walls of flowers as they open and senesce (O’Donoghue *et al.*, 2009; O’Donoghue and Sutherland, 2012).

Another category highly enriched in petal tissues was ‘response to light’. Involvement of light as an extreme light condition (i.e. darkness) in regulating plant senescence has been reported in a number of studies (Weaver and Amasino, 2001; Buchanan-Wollaston *et al.*, 2005; van der Graaff *et al.*, 2006). More recent research has now revealed how classic light signalling connects to senescence in two different *Arabidopsis* tissue systems: leaves (Song *et al.*, 2014; Sakuraba *et al.*, 2014) and inflorescences (Trivellini *et al.*, 2012).

### Network of transcriptional regulation involved in the control of developmentally regulated flower senescence

Our aim was to use expression profiling to identify genes associated with ageing in the different organs of the flower. Gene transcripts that are present in tissue only at a specific developmental stage and gene transcripts that are present in higher abundance in these tissues are valuable for understanding the biological and metabolic processes during the flower ageing process. Using annotated sequences as an experimental filter and query input, we identified candidate genes for triggering and signalling (Ca and ROS signals, kinases and G proteins), transcriptional regulation, coordination (phytohormones and cell wall modification), and execution of the ageing process (proteases, nucleases, and autophagy) as defined by Van Hautegem *et al.* (2015).

### Triggering and signalling

#### Light stimuli and circadian clock

**Fig. 4.**
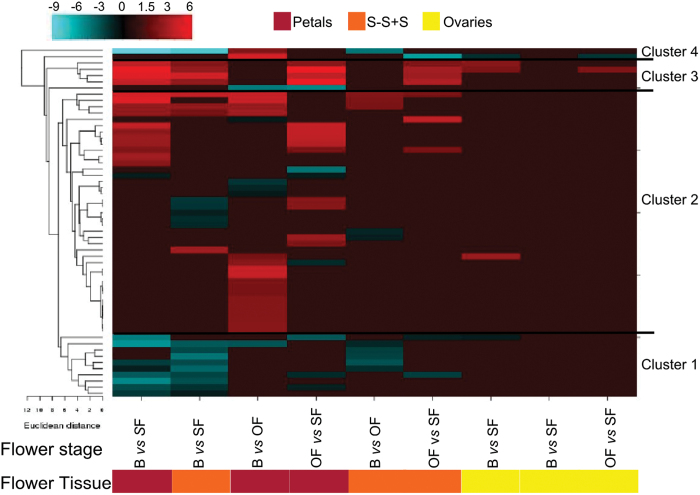
Transcript abundance changes and cluster analysis for the light signalling gene set that was differentially expressed among flower tissues and stages. The cluster analysis for each group of genes was performed using hierarchical clustering with average linkage and Euclidian distance measurement. Rows represent differentially expressed genes, and columns represent the tissue and developmental stages of flowers considered. The log-2 ratio values of significantly regulated transcripts were used for hierarchical clustering analysis. The transcript ID and annotation of each gene are listed in Supplementary Table S4, and the cluster numbers are listed on the left. Red and blue indicate upregulation and downregulation, respectively.

Many flowers and in particular ephemerals such as hibiscus open in the morning and start to close and senesce before twilight (Trivellini *et al.*, 2011*a*, *b*). In general, the opening and subsequent closure of ephemeral corollas appear to be linked to dual systems: one controlled by a circadian clock and the other by the effect of light (Bai and Kawabata, 2016). However, it is not known how the light perception signals are transduced to control floral opening and longevity. Four gene clusters associated with light signalling were identified in the hibiscus flower tissues using a transcript ratio of >2 as a filter and hierarchical clustering (Fig. 4, Supplementary Table S4). Cluster 1 contained 10 transcripts mainly induced in petals and the S-S+S of the SF (Fig. 4). Among them was a gene encoding phytochrome kinase substrate 1 (PKS1), which controls phototropin and phytochrome‐mediated growth responses (Lariguet *et al.*, 2006). Three transcripts were also detected that encode a phytochrome A-associated F-box protein (EID1), which functions as a negative regulator in phytochrome A (phyA)-specific light signalling (Marrocco *et al.*, 2006), and three other transcripts were identified that encode chlorophyll a/b binding proteins (CP29.3, CP4, and 26). Clusters 2 and 3 identified transcripts associated with light-induced modifications of both the actin and microtubule cytoskeleton, such as myosin light polypeptide and dynein light chain, which may represent a very early event triggering flower opening and senescence (Galvão and Fankhauser, 2015). PHOTOTROPIN1 (PHOT1) is a blue light receptor which in Arabidopsis leaves declines during senescence (Łabuz *et al.*, 2012). However, in hibiscus, transcript abundance of *PHOT1* was elevated in the S-S+S and petals of SF (Fig. 4, Supplementary Table S4). Seven chlorophyll a/b binding proteins were also elevated in these tissues; conversely, the transcript abundance of eight early light-inducible proteins (ELIPs) was suppressed. PHOT1 has been reported to be the light receptor for the circadian clock (Ahmad *et al.*, 2002; Litthauer *et al.*, 2015), and it has been established that the ELIPs and the light-harvesting chlorophyll a/b binding proteins are under circadian clock control (McClung *et al*., 2006). This suggests that the development of hibiscus flowers is accurately synchronized by both light and an endogenous clock. The role of the circadian clock in modulating flower opening and senescence was previously shown in *Mirabilis jalapa*, where the opening and senescence of flowers was found to be regulated by a circadian rhythm in which light input was sensed by the phytochrome A pathway (Xu *et al.*, 2007). Overall, the data suggest that the very rapid senescence programme of the ephemeral hibiscus flower involves light/circadian rhythm signalling events.

#### Calcium signalling

Calcium (Ca^2+^) is a key regulator of many processes in plants (Batistiĉ and Kudla, 2012). The Ca^2+^ signal is transduced in plants through the action of specific sensors/transporters. These include calmodulin (CaM), calmodulin-like proteins (CaMLs), Ca^2+^-dependent protein kinases, calcineurin B-like proteins (CBLs), and CBL-interacting kinases (CIPKs). A total of 238 unigenes were found to be related to Ca^2+^ signalling and show different levels of expression (Supplementary Table S5). The abundance of putative gene transcripts of CaM and CaML was higher in petals of SF. CaML transcripts were also more highly abundant in S-S+S tissues. In general, transcripts related to CaM-binding proteins were more abundant in both petals and S-S+S of OF and SF tissues, and CIPK transcripts were in general more abundant in petals and S-S+S during the OF and SF developmental stages. CBLs and CIPKs have been reported to be involved in abiotic stress responses (Albrecht *et al.*, 2003) and responses to ABA (Batistiĉ and Kudla, 2012). The abundance of several transcripts related to the CaM-binding protein calreticulin was higher in buds, suggesting the activation of independent transcriptional regulators at different developmental stages. Overall, however, these results strongly suggest that the Ca^2+^ signalling occurring in the different cellular compartments of the different floral parts is an important component of the flower senescence programme.

#### ROS metabolism

Flower senescence is accompanied by the production and accumulation of ROS. ROS levels tend to increase during senescence, but it is not yet clear how this is connected to developmental senescence (van Doorn and Woltering, 2008; Salleh *et al.*, 2016). In hibiscus, transcripts encoding enzymes committed to ROS control were enriched (Supplementary Table S6). There were significant changes in transcript abundance for 28 ROS signalling-related genes. Transcript abundance of the cytosolic form of ascorbate peroxidase (APX), the key enzyme in the ascorbate-glutathione pathway (a major plant antioxidant defence pathway; Halliwell, 2006), was higher in petals at B stage. Transcript abundance for a putative peroxisomal form, APX3, was higher in petals and S-S+S of SF. Transcript changes of monodehydroascorbate reductase (MDHAR) were similar to those of APX, being more abundant in petals at B stage compared to OF stage. Higher numbers of transcripts were also found in petals and S-S+S of SF compared with B. These findings indicate that the protective systems against ROS damage are still active in these organs as they senesce. In the ovary, MDHAR was upregulated in B and OF, whereas it was not expressed during senescence.

Together with the ascorbate-glutathione cycle, other enzymatic systems act to control ROS accumulation. Glutathione peroxidases (GPX) catalyse the reduction of H_2_O_2_ or organic hydroperoxides to water, and have been found in almost all kingdoms of life (Passaia *et al.*, 2013). Gene transcripts encoding GPX were more abundant in petals and S-S+S of SF than B, whereas the GPX transcript was more highly abundant in B compared to OF (in petals only). Glutaredoxins (GLR) are a family of enzymes capable of being oxidized by different substrates (among those, dehydroascorbic acid) and that use glutathione as a cofactor (Delorme-Hinoux *et al.*, 2016). The gene transcripts encoding for different forms of GLR were highly abundant in petals and in S-S+S of OF. Higher transcripts quantities were found for genes encoding peroxiredoxins in petals and S-S+S, particularly in B. These peroxidases have been reported to be involved in modulating redox signalling during development and adaptation (Dietz *et al*., 2003).

### Transcriptional regulation

#### Transcription factors

**Fig. 5.**
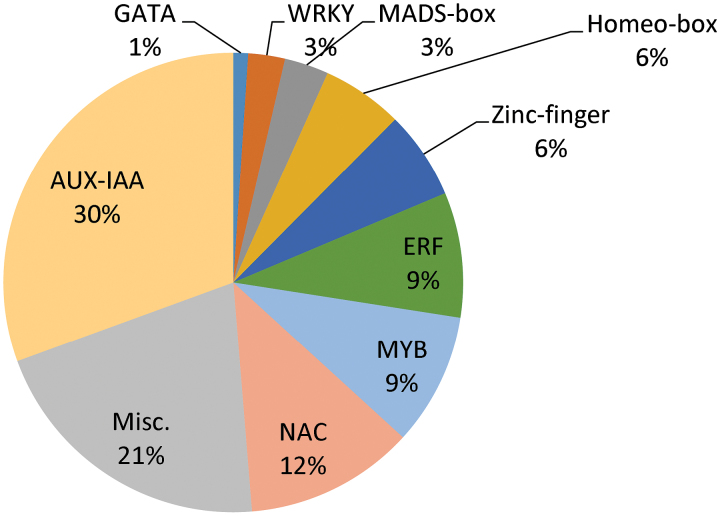
Most represented TF families found in the transcriptome of hibiscus. Number of transcripts related to each specific family are clustered and shown as the percentage of the total number of putative TFs that were differentially expressed.

Analysis of the hibiscus transcriptome identified more than 190 putative genes encoding for transcription factors (TFs) (Supplementary Table S7). Most were clustered into nine families: IAA, NAC, ERF, MYB, WRKY, MADS-box, Zinc-finger, homeobox, and GATA (Fig. 5). The most highly represented family was AUX-IAA (30.5%). The transcripts of the majority of AUX-IAA genes had a significantly higher abundance in petals and in S-S+S than in the ovary. In general, their transcript abundance was higher in B and OF stages than in the SF stage. The NAC family was among those differentially expressed in petals, S-S+S, and ovary. In general, most of the NAC transcripts had higher abundance in the SF stage than at the B or OF stage. This result is consistent with previous data reported by Wagstaff *et al.* (2009) in Arabidopsis, in which the NAC family was the first of the TF families to be up-regulated in all senescent tissues studied. The role of AP2/ERF TFs in senescence is now well established (Rogers, 2013; Wagstaff *et al.*, 2009). In our study with hibiscus, around 9% of the TFs were related to ethylene response. The ethylene-responsive TFs were detected mainly in petals and S-S+S, and the majority were more abundant in the SF. These included ERF3, EREB1, and EREB2. ERF3 was previously reported to be particularly associated with flower senescence in petunia (Liu *et al.*, 2010). Approximately 9% of the differentially expressed TFs belonged to the MYB family. However, their expression varied significantly among the tissues and developmental stages. The most representative found were associated with colour development changes (MYB308, MYB305, MYB90) and/or to stress response (MYB75) (Ambawat *et al.*, 2013). As previously reported (Wagstaff *et al.*, 2009), transcripts of WRKY TFs had a higher abundance in the petal and S-S+S tissues of the SF. WRKY53, which is a key TF for controlling leaf senescence, was not identified in the SF tissue of hibiscus. It was also not upregulated in senescent petals in previous studies (Rogers, 2013; Wagstaff *et al.*, 2009), which suggests a minor role for this TF in flower senescence.

### Coordination

#### Hormones

Generally plant development is coordinated by hormones. Ethylene precursor (ACC) applied exogenously has previously been shown to accelerate senescence of the hibiscus flower (Trivellini *et al.*, 2011*b*). The current transcriptome profiling reveals that the accelerated senescence is caused by enhanced signalling that would naturally occur via transcriptional upregulation of the ethylene biosynthetic pathway during flower development. Transcripts associated with biosynthetic genes (ACC oxidases and synthase) and ethylene signalling were significantly differentially regulated among flower stages and tissues (Supplementary Table S8). Ethylene is involved in a number of essential processes during flower development, including sexual determination (Yamasaki *et al.*, 2003), petal senescence (van Doorn and van Meeteren, 2003; van Doorn and Woltering, 2008), floral organ abscission (Patterson and Bleecker, 2004; Butenko *et al.*, 2006), and petal development during the process of flower opening (Ma *et al.*, 2006; Xue *et al.*, 2008). While the results of the present study are consistent with our previous publications, it still cannot be ignored that even in the presence of an ethylene biosynthesis inhibitor, AOA (Trivellini *et al.*, 2011*a*), or an ethylene action inhibitor, 1 MCP (Trivellini *et al.*, 2011*b*), the senescence of hibiscus flowers is only delayed by about 3–5h. This suggests that even though ethylene regulates hibiscus flower senescence, the process also occurs in an ethylene-independent manner.

Few genes involved in cytokinin metabolism were identified in the hibiscus flower (Supplementary Table S9). A transcript encoding a protein involved in degradation of cytokinins, cytokinin oxidase/dehydrogenase, was lower in abundance in petals at B stage, and transcripts encoding the type-A response regulator ARR8, which is a negative regulator of CK signalling, had a higher abundance in the ovary at SF stage. Combined with the differential regulation of cytokinin-binding proteins mainly in B tissues, this suggests that cytokinin signalling might be involved in an earlier flower stage. These finding are in line with the higher sensitivity of flower bud to exogenous cytokinin treatment, which also accelerates flower development (Hernández *et al.*, 2015) and delays senescence of the petunia flower (Trivellini *et al.*, 2015*a*).

Transcripts encoding the rate-limiting ABA biosynthesis enzyme 9-cis-epoxycarotenoid dioxygenases (NCED) were higher in abundance in petals and S-S+S of OF versus SF. Moreover, a xanthoxin dehydrogenase gene that encodes an intermediate in the biosynthesis of ABA was more than 2-fold higher both in the S-S+S and ovary of SF. Interestingly, in line with the increase of ABA content previously reported in hibiscus flower tissues (Trivellini *et al.*, 2011*a*), a transcript that encodes the *ABA4* gene, which is involved in neoxanthin synthesis, was higher in abundance in petals and S-S+S of SF, suggesting that biosynthesis in response to ageing must occur mainly via neoxanthin isomer precursors (North *et al.*, 2007). This suggests enhanced ABA signalling, possibly via increased ABA biosynthesis through the carotenoid pathways, facilitates the progression of flower ageing in hibiscus (Supplementary Table S10).

Several genes related to auxin signalling and synthesis were differentially regulated in hibiscus tissues (Supplementary Table S11). This suggests that auxin production in specific tissues has a vital role during flower development (Stepanova *et al.*, 2008). Genes encoding the nodulin MtN21 family protein showed the greatest decline in transcript abundance in the young versus senescing tissue. Homologues of nodulin genes are found in the genomes of several plants that are unable to nodulate, highlighting an ancestral role in plant physiology (Denancé *et al.*, 2014). An MtN21 protein-encoding gene has recently been proposed to act as a vacuolar auxin export facilitator in isolated Arabidopsis vacuoles (Ranocha *et al.*, 2013), as a bidirectional amino acid transporter (Ladwig *et al.*, 2012), and as a tonoplast-localized protein required for proper secondary cell wall formation (Ranocha *et al.*, 2010). Combined with the induction of the auxin receptor, auxin-binding protein, and the several auxin regulated and induced proteins (AUX/IAA), auxin signalling might be involved in flower opening and senescence possibly by facilitating cell wall remodelling (Paque *et al.*, 2014).

Gibberellin (GA)-related gene transcripts differentially accumulated in the different tissues and flower stages (Supplementary Table S12). GAs control cell proliferation and expansion. When GA accumulates and binds to the GIBBERELLIN INSENSITIVE DWARF1 (GID1) GA receptors, DELLA proteins are degraded, allowing the expression of downstream genes (Claeys *et al.*, 2014). The inactivation of GA is usually catalysed by GA2OX, whose transcript in hibiscus was substantially lower in both petals and S-S+S of B. The *GA2OX* gene transcripts were mainly induced in OF stages, supporting the view that its protein was formed to inactivate GA and stop further expansion of the petal tissues. The steps linking transcriptional activation to physiological responses in GA signalling are carried out by *GAST*-like genes, which encode small proteins with a conserved cysteine-rich domain (Rubinovich and Weiss, 2010). The role of these proteins in flower development is not yet clear. Interestingly, *GAST*-like genes (*GASA4* and *GASA1* genes) were among the GA-signalling genes most strongly induced in petals and S-S+S of B, OF, and SF (Supplementary Table S12). In strawberry (*Fragaria × ananassa*), overexpression of *FaGAST* inhibits cell elongation, causing delayed growth (de la Fuente *et al.*, 2006). Another *GAST1* homologous gene in gerbera (*Gerbera hybrida*) is induced late in the development of the corolla as cellular expansion ceases (Kotilainen *et al.*, 1999). Thus, it is possible that GA induces the transcription of various *GAST1*-like genes and, as reported by Rubinovich and Weiss (2010), their encoded proteins reduce or oxidize specific targets, such as cell wall proteins (that are tightly associated with the cell wall matrix; Ben-Nissan *et al.*, 2004), to control the flowering process.

#### Cell wall-related activity

**Fig. 6.**
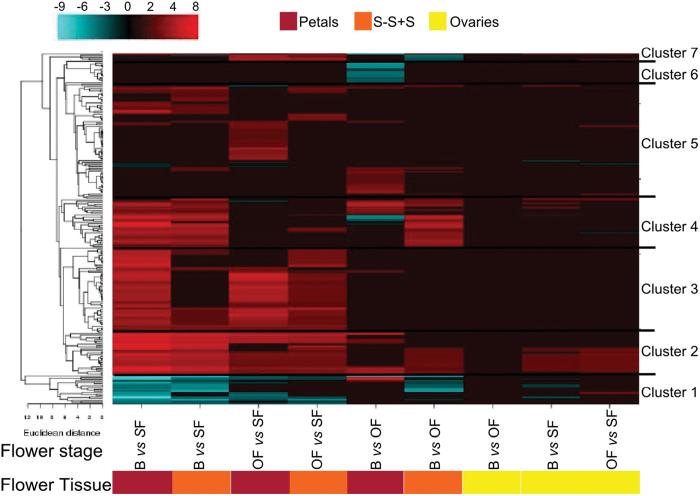
Transcript abundance changes and cluster analysis of the cell wall-modifying gene set that was differentially expressed among flower tissues and stages. The cluster analysis for each group of genes was performed using hierarchical clustering with average linkage and Euclidian distance measurement. Rows represent differentially expressed genes, and columns represent the tissue and developmental stages of flowers considered. The log-2 ratio values of significantly regulated transcripts were used for hierarchical clustering analysis. The transcript ID and annotation of each gene are listed in Supplementary Table S13, and the cluster numbers are listed on the left. Red and blue indicate upregulation and downregulation, respectively.

Downstream of hormonal signalling there are changes in gene transcript abundance of a very large number of genes whose proteins are associated with cell wall activities (Supplementary Table S13). This supports the finding of O’Donoghue and Sutherland (2012) that flower development involves substantial changes in cell wall structure. Hierarchical cluster analysis classified the cell wall gene set into seven distinct groups (Fig. 6). The first and seventh clusters include 25 genes encoding mainly xyloglucan endotransglycosylases (XETs). In cluster 1, these genes were significantly downregulated in young versus senescent tissues, whereas in cluster 7 these families showed an opposite trend (upregulation in mature/senescent versus young tissues) predominately in petals and S-S+S tissues. Cluster 2 contained 28 members belonging to the expansin family and these genes were more highly expressed in all young/mature tissues than in senescent tissues. Cluster 3 predominantly contained pectate lyase transcripts that had a similarly high abundance in the petals and S-S+S tissues of B and OF. Interestingly, in cluster 4 were transcripts encoding endo-1,4-β-glucanase, fasciclin-like arabinogalactan proteins, and expansins that had higher abundance principally in young versus mature/senescent tissues. Tissue- and stage-specific expression of miscellaneous cell wall-modifying enzymes characterized cluster 5. In cluster 6, among the 14 transcripts strongly downregulated in petals of B versus OF, three of them encoded expansins, two of them XETs, and the majority endoglucanases. These transcriptome changes are consistent and highlight that cell wall metabolism has a key role in flower opening and senescence. In fact, in closed flower buds and during anthesis, genes encoding expansins and XETs, which are involved in cell wall loosening, are expressed in carnation (Harada *et al.*, 2011) and *Eustoma* (Ochiai *et al.*, 2013). Moreover, endo-1,4-β-glucanases are closely associated with cell elongation and fruit ripening (Yang *et al.*, 2016), pectate lyases are involved in pollen development and/or function and fruit softening (Palusa *et al.*, 2007), and fasciclin-like arabinogalactan proteins were recently proposed to play an important role in the plant sexual reproductive process (Pereira *et al.*, 2014). The downregulated groups were dominated by XET genes, which previously have been reported to be expressed throughout all stages of cell wall disassembly, and during abscission (Singh *et al.*, 2011; Singh *et al.*, 2013).

**Fig. 7.**
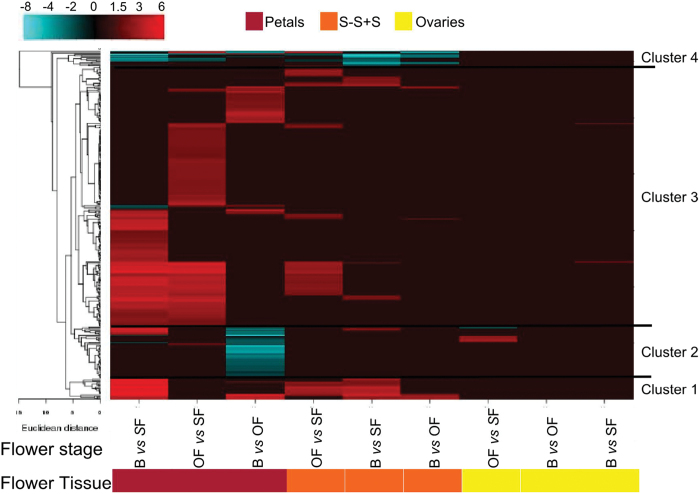
Transcript abundance changes and cluster analysis of the aquaporin gene set that was differentially expressed among flower tissues and stages. The cluster analysis for each group of genes was performed using hierarchical clustering with average linkage and Euclidian distance measurement. Rows represent differentially expressed genes, and columns represent the tissue and developmental stages of flowers considered. The log-2 ratio values of significantly regulated transcripts were used for hierarchical clustering analysis. The transcript ID and annotation of each gene are listed in Supplementary Table S14, and the cluster numbers are listed on the left. Red and blue indicate upregulation and downregulation, respectively.

Cellular expansion and collapse during flower development and ageing processes are regulated not only by cell wall-modifying enzymes but also transcellular and transmembrane water transport. Cut roses are a good example for illustrating this as the flowers will not open if water transport is impaired by a blockage in their basal stem (van Doorn and van Meeteren, 2003). Transcriptional regulation of aquaporins during anthesis and senescence showed massive differences, with more than 300 genes expressed mainly in petals of B and PF and then in S-S+S tissue (Fig.7; Supplementary Table S14). Aquaporins are highly regulated channels controlling plant–water relations in plants (Chaumont and Tyerman, 2014). Examples of aquaporins are plasma membrane intrinsic proteins and tonoplast intrinsic proteins. Interestingly, the role of ethylene in flower opening may also be expanded to its effect on aquaporins. It was previously shown that ethylene inhibits petal expansion of roses at least partially by suppressing a specific aquaporin (Ma *et al.*, 2008). Moreover, considering that the transcription of aquaporins is regulated by the circadian clock (Chaumont and Tyerman, 2014), it is likely that circadian clock regulation of flower development occurs in part via aquaporins. This condition might be supported by the considerable differential regulation of aquaporin gene expression among different flower developmental stages. In hibiscus, it has been shown that flower senescence entrains circadian rhythms via endogenous oscillations in sugars (Trivellini *et al.*, 2011*c*). This opens the possibility that variation in sugar may be important in entrainment of the clock in the flower petals and regulation of aquaporins. Finally, these results suggest that in hibiscus flowers the petal movements occurring during flower opening and closing might be facilitated by aquaporins, which are important for motor cell dynamics.

### Execution

Flower senescence is a highly regulated developmental process that leads the organ to a rapid and irreversible programmed cell death during the last stages (Van Hautegem *et al.*, 2015). Van Doorn *et al*. (2011) established nomenclature for the complex cell death mechanisms in plants and proposed unified criteria for their definition. Recent studies have suggested that autophagy is one of the main mechanisms of degradation and remobilization of macromolecules, and it appears to play an important role in petal senescence (Yamada *et al.*, 2009; Shibuya *et al.*, 2013). Autophagy is a type of programmed cell death first identified in animal cells that is characterized by the appearance of vacuole-like vesicles involved in engulfment of the cytoplasm and its subsequent degradation (van Doorn *et al.*, 2011). Seven transcripts encoding autophagy genes (*ATG8c*, *ATG8f*, and *ATGg*; Supplementary Table S15) were strongly induced in senescing petals and S-S+S tissues. During hibiscus flower senescence, the nitrogen, phosphorus, potassium, and micro-nutrient content of petals and S-S+S is significantly reduced, supporting a possible role of autophagy in the degradation and remobilization of macromolecules during flower ageing (Trivellini *et al.*, 2011*c*). These data support the previously documented role for autophagy in petal senescence (Yamada *et al.*, 2009; Shibuya *et al.*, 2013), pointing out also its role in senescing S-S+S as a major nutrient recycling mechanism in plants as suggested by Coll *et al.* (2014). Many of the senescence upregulated genes that have been identified from petals and S-S+S tissues encode nucleases and proteases, which are catabolic enzymes involved in the breakdown of nucleic acids and proteins, respectively (Diaz-Mendoza *et al.*, 2014; Sakamoto and Takami, 2014) (Supplementary Table S15). Moreover, the transcriptional signatures of flower ageing in hibiscus share common features with the conserved core of developmentally regulated programmed cell death signatures in plants recently reported by Olvera-Carrillo *et al.* (2015). In fact, transcripts for ribonuclease 3, calcium-dependent nuclease 1, metacaspase, cysteine proteases, aspartic proteases, cysteine endopetidases, and vacuolar processing enzymes were more abundant in senescing petals and S-S+S, defining the transcriptional regulation that leads to the rapid execution of cell death observed in many systems (Coll *et al.*, 2014; Hatsugai *et al.*, 2015; Mochizuki-Kawai *et al.*, 2015; Olvera-Carrillo *et al.*, 2015).

## Conclusions

**Fig. 8.**
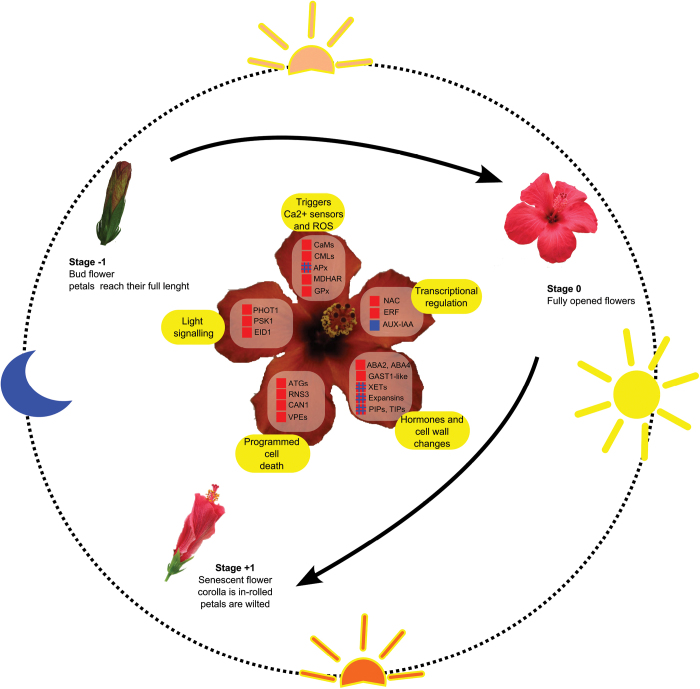
Overview of flower senescence regulation. Red and blue indicate gene upregulation and downregulation, respectively.

This work has highlighted the transcriptional signature of flower-tissue senescence in hibiscus (Fig. 8). The development of hibiscus flowers (bud stage, opening, and senescence) is perfectly synchronized with light. All the flowers that open within a day open and senesce at exactly the same time. This suggests that the circadian clock is an important key regulator of hibiscus flower development. This conclusion is supported by the significant enrichment of light related gene transcripts (including genes of the phyA pathway, ELIPs, and chlorophyll-binding proteins) found in the functional category analysis, and of the phototropin PHOT1 blue light receptor, which was mainly induced during the hibiscus senescence stage in petals and the S-S+S complex.

The life span of the ephemeral hibiscus flower may be controlled in part by a light‐sensing system, which determines alteration of the cytoskeleton to allow flower opening and then petal in-rolling during senescence. Some common themes between senescence of leaves and flowers have emerged, including the production and accumulation of ROS caused by upregulation of protection systems against ROS damage, and the elaborated activation in senescing tissues of specific Ca^2+^ sensors like CaM and CaMLs. Various key TFs have been identified and the families most represented were IAA-AUX, NAC, and ERF. While the involvement of NAC and ERF in senescence has been documented previously, the link between auxin-responsive transcriptional regulators and flower senescence needs to be further investigated. Nevertheless, this work provided additional information on their activation during flower development, and will help enable hypotheses to be developed for the role of these TFs at the early stage of flowering compared to senescence. Several cell wall-modifying enzymes, including XETs, expansins, pectate lyases, endo-1,4-β-glucanase, and fasciclin-like arabinogalactan, were differentially regulated in senescing floral tissue, suggesting that cell wall metabolism is key for flower opening and senescence. The transcriptome information provided potential molecular identification of the aquaporins that are differentially regulated to control the macroscopic petal opening and subsequent senescence-associated collapse. Finally, gene targets were identified that are likely responsible for the irreversible senescence (programmed cell death) that occurs in the hibiscus flower, for example, genes encoding aspartic and cysteine proteases, vacuolar processing enzymes, nucleases, and autophagy. Together, these results provide useful clues on what should be investigated further to reveal the unknown pathways of flower programmed death.

## Supplementary data

Supplementary data are available at *JXB* online.

Table S1. Design of the built microarray chips.

Table S2. All differentially expressed transcripts among separate floral tissues (petals, S-S+S and ovary) of the bud vs open flower, bud vs senescent flower, and opened flower vs senescent flower.

Table S3. Enrichment analysis using DAVID functional annotation clustering tools.

Table S4. List of selected ‘light signalling’ genes with the relative expression levels (FC) in Hibiscus flower tissues (petal, S-S+S, ovary) at different developmental stages (B, OF, and SF).

Table S5. List of selected ‘calcium signalling’ genes with the relative expression levels (FC) in Hibiscus flower tissues (petal, S-S+S, ovary) at different developmental stages (B, OF, and SF).

Table S6. List of selected ‘ROS metabolism’ genes with the relative expression levels (FC) in Hibiscus flower tissues (petal, S-S+S, ovary) at different developmental stages (B, OF, and SF).

Table S7. List of selected ‘transcription factor’ genes with the relative expression levels (FC) in Hibiscus flower tissues (petal, S-S+S, ovary) at different developmental stages (B, OF, and SF).

Table S8. List of selected ‘ethylene signalling pathway’ genes with the relative expression levels (FC) in Hibiscus flower tissues (petal, S-S+S, ovary) at different developmental stages (B, OF, and SF).

Table S9. List of selected ‘cytokinin signalling pathway’ genes with the relative expression levels (FC) in Hibiscus flower tissues (petal, S-S+S, ovary) at different developmental stages (B, OF, and SF).

Table S10. List of selected ‘ABA signalling pathway’ genes with the relative expression levels (FC) in Hibiscus flower tissues (petal, S-S+S, ovary) at different developmental stages (B, OF, and SF).

Table S11. List of selected ‘auxin signalling pathway’ genes with the relative expression levels (FC) in Hibiscus flower tissues (petal, S-S+S, ovary) at different developmental stages (B, OF, and SF).

Table S12. List of selected ‘gibberellin signalling pathway’ genes with the relative expression levels (FC) in Hibiscus flower tissues (petal, S-S+S, ovary) at different developmental stages (B, OF, and SF).

Table S13. List of selected ‘cell wall-modifying enzyme’ genes with the relative expression levels (FC) in Hibiscus flower tissues (petal, S-S+S, ovary) at different developmental stages (B, OF, and SF).

Table S14. List of selected ‘aquaporin’ genes with the relative expression levels (FC) in Hibiscus flower tissues (petal, S-S+S, ovary) at different developmental stages (B, OF, and SF).

Table S15. List of selected ‘cell death execution’ genes with the relative expression levels (FC) in Hibiscus flower tissues (petal, S-S+S, ovary) at different developmental stages (B, OF, and SF).

Supplementary Data
