# Supplementary material for: Spatial and temporal transcriptome changes occurring during flower opening and senescence of the ephemeral hibiscus flower, Hibiscus rosa-sinensis
Source: J Exp Bot. 2016 Sep 3;67(20):5919–31. doi: 10.1093/jxb/erw295 (PMC5091337; doi:10.1093/jxb/erw295)
Supplement: Supplementary Data [file supp_67_20_5919__index.html]

Spatial and temporal transcriptome changes occurring during flower opening and senescence of the ephemeral hibiscus flower, Hibiscus rosa-sinensis — Spatial and temporal transcriptome changes occurring during flower opening and senescence of the ephemeral hibiscus flower, Hibiscus rosa-sinensis — Supplementary Data 

# Spatial and temporal transcriptome changes occurring during flower opening and senescence of the ephemeral hibiscus flower, *Hibiscus rosa-sinensis*

## Supplementary Data

Data files

- supplementary\_table\_S1.docx - Supplementary Data
- supplementary\_table\_S2.xls - Supplementary Data
- supplementary\_table\_S3.xlsx - Supplementary Data
- supplementary\_table\_S4.xlsx - Supplementary Data
- supplementary\_table\_S5.xlsx - Supplementary Data
- supplementary\_table\_S6.xlsx - Supplementary Data
- supplementary\_table\_S7.xlsx - Supplementary Data
- supplementary\_table\_S8.xlsx - Supplementary Data
- supplementary\_table\_S9.xlsx - Supplementary Data
- supplementary\_table\_S10.xlsx - Supplementary Data
- supplementary\_table\_S11.xlsx - Supplementary Data
- supplementary\_table\_S12.xlsx - Supplementary Data
- supplementary\_table\_S13.xlsx - Supplementary Data
- supplementary\_table\_S14.xlsx - Supplementary Data
- supplementary\_table\_S15.xlsx - Supplementary Data
