# Supplementary material for: Spatial and temporal transcriptome changes occurring during flower opening and senescence of the ephemeral hibiscus flower, Hibiscus rosa-sinensis
Source: J Exp Bot. 2016 Sep 3;67(20):5919–31. doi: 10.1093/jxb/erw295 (PMC5091337; doi:10.1093/jxb/erw295)
Supplement: Supplementary Data [file supp_erw295_supplementary_table_S1.docx]

Suppl. table 1. Hybridazation scheme of microarray 4 x 44 K Agilent. In each microarray contains 4 slides and in each slide the RNA samples used are reported.

| **Microarray 1** |  |  |  |  |  |  |
| --- | --- | --- | --- | --- | --- | --- |
| ovary (B) 13 |  | ovary (OF) 15 |  | ovary (B) 13 |  |  |
| Vs |  | vs |  | vs |  | DYE SWAP |
| ovary (OF) 15 |  | ovary (SF) 17 |  | ovary (SF) 17 |  |  |
|  |  |  |  |  |  |  |
| **Microarray 2 (2nd biological replication)** |  |  |  |  |  |  |
| ovary (B) 14 |  | ovary (OF) 16 |  | ovary (B) 14 |  |  |
| vs |  | vs |  | vs |  | DYE SWAP |
| ovary (OF) 16 |  | ovary (SF) 18 |  | ovary (SF) 18 |  |  |
|  |  |  |  |  |  |  |
| **Microarray 3** |  |  |  |  |  |  |
| s-s+s (B) 7 |  | s-s+s (OF) 9 |  | s-s+s (B) 9 |  | petal (B) 1 |
| vs |  | vs |  | vs |  | vs |
| s-s+s (OF) 9 |  | s-s+s (SF) 11 |  | s-s+s (SF) 11 |  | petal (OF) 3 |
|  |  |  |  |  |  |  |
| **microarray 4 (2nd Biological replication)** |  |  |  |  |  |  |
| s-s+s (B) 8 |  | s-s+s (OF) 10 |  | s-s+s (B) 8 |  | petal (OF) 3 |
| vs |  | vs |  | vs |  | vs |
| s-s+s (OF) 10 |  | s-s+s (SF) 12 |  | s-s+s (SF) 12 |  | petal (SF) 5 |
|  |  |  |  |  |  |  |
| **Microarray 5** |  |  |  |  |  |  |
| petal (B) 2 |  | petal (OF) 4 |  | petal (B) 2 |  | petal (B) 1 |
| vs |  | vs |  | vs |  | vs |
| petal (OF) 4 |  | petal (SF) 6 |  | petal (SF) 6 |  | petal (SF) 5 |

**Samples legend:**

Bud Petals (BP1; BP2) = (1 ,2)

Open flower petals (OFP1; OFP2) = (3,4)

Senescent flower petals (SFP1; SFP2)= (5, 6)

Bud s-s+s (BS1; BS2)= (7, 8)

Open flower s-s+s (OFS1; OFS2)= (9, 10)

Senescent flower s-s+s (SFS1; SFS2)= (11, 12)

Bud ovary (BO1; BO2)= (13, 14)

Open flower ovary (OFO1; OFO2)= (15, 16)

Senescent flower ovary (SFO1; SFO2)= (17, 18)
